# New Insight into the Microstructure Changes and Molecular Mobility of Polyamides Exposed to H_2_S Scavengers

**DOI:** 10.3390/polym17121634

**Published:** 2025-06-12

**Authors:** Marina Perassoli de Lazari, Antonio Henrique Monteiro da Fonseca Thomé da Silva, Rodrigo Henrique dos Santos Garcia, Sylvia Correa dos Santos Teixeira, João Eduardo de Oliveira, Érica Gervasoni Chaves, Luiz Antônio de Oliveira Nunes, Hercílio de Angeli Honorato, Sonia Maria Cabral de Menezes, Aline Pinde Lima, Luiz Silvino Chinelatto Junior, Eduardo Ribeiro de Azevedo

**Affiliations:** 1 Instituto de Física de São Carlos, Universidade de São Paulo, 400 Parque Arnold Schimidt, São Carlos 13566-590, SP, Brazilluizant@ifsc.usp.br (L.A.d.O.N.); 2 PETROBRAS/CENPES, Av. Horácio Macedo 950, Rio de Janeiro 21941-915, RJ, Brazilhercilio.deangeli@petrobras.com.br (H.d.A.H.); alineplima@petrobras.com.br (A.P.L.); lsilvino@petrobras.com.br (L.S.C.J.); 3 Departamento de Engenharia Mecânica, Universidade Federal Fluminense–UFF/PGMEC, Niterói 24210-240, RJ, Brazil; 4 Instituto de Física, Universidade de São Paulo, Rua do Matão 1371, Cidade Universitária, São Paulo 05508-090, SP, Brazil; scdemenezes@yahoo.com

**Keywords:** ^1^H time-domain NMR, solid state ^13^C NMR, infrared spectroscopy, polyamide degradation, H_2_S scavenger, plasticizer exudation

## Abstract

Polyamides (PAs) are widely used as barrier materials in offshore oil and gas (O&G) equipment due to their mechanical strength and chemical resistance. However, long-term exposure to hydrogen sulfide scavengers (H_2_S-SCVs) may significantly affect their physicochemical properties. Previous studies using thermal analysis and ^1^H time-domain NMR (^1^H TD-NMR) suggest that ethoxylated H_2_S-SCVs impose molecular constraints, increasing the glass transition temperature (T_g_) and reducing chain mobility above T_g_. The present study builds upon these findings using a multi-technique analytical approach, including FTIR, Raman, ^1^H DQ-TD-NMR, and ^13^C solid-state NMR (ssNMR), to provide a more comprehensive understanding of the molecular alterations in PA materials. The results clearly demonstrate that H_2_S-SCV exposure leads to the progressive exudation of plasticizers from the PA matrix. This plasticizer loss is a key factor contributing to the observed shift in Tg and the reduction in molecular mobility. ^1^H DQ-TD-NMR data confirmed an increase in the density of dynamically constrained chains over time and allowed for the characterization of heterogeneity in these constraints throughout the PA matrix. Moreover, ^13^C ssNMR spectra revealed the presence of immobilized H_2_S-SCV chemical groups within the polymer matrix, strongly supporting the early statement that the mobility constraints observed in ^1^H DQ-TD-NMR are associated with the formation of crosslinks induced by the H_2_S-SCV: H_2_S-SCV acts as a crosslinking agent. Taken together, our findings indicate that both plasticizer loss and H_2_S-SCV-induced crosslinking contribute significantly to the microstructural evolution of PAs when exposed to ethoxylated H_2_S-SCVs, offering important insights into their degradation mechanisms and long-term behavior in aggressive operational environments.

## 1. Introduction

Polymers are key materials in numerous contemporary technological applications. For instance, active polymer layers constitute the core of various organic electronic semiconductor devices, including polymer-based Light-Emitting Diodes (LEDs), transistors, flexible solar cells, and neuromorphic memories [1,2]. Despite the widespread use of active polymers in advanced applications, their significant utility remains closely tied to conventional polymers. These polymers find applications in textiles, construction, plastics, rubber, oil and gas, and various other industrial sectors.

Even in more traditional polymer applications, significant technological advancements have been made in materials production. These advancements have led to remarkable improvements in mechanical strength, durability, flexibility, permeability to liquids and gases, and resistance to degradation, among other properties. It is worth noting that each improvement is tailored to the specific application and type of polymer, with enhanced resistance to degradation being a common focus across diverse applications.

In a broad context, polymer degradation is linked to the interaction of polymer chains with external agents, resulting in changes to the chemical composition, microstructure, morphology, or dynamics of the polymer chains, thereby affecting desired macroscopic properties. Consequently, comprehending the degradation mechanism and its connection with the macroscopic properties of interest is essential for developing strategies that either mitigate polymer degradation or prevent conditions that promote the degradation process.

Polymeric materials play a widespread role in the oil and gas (O&G) industry, particularly within the infrastructure used in offshore production [3]. In these applications, one of their primary functions is in the design of flexible pipelines, where polymer layers not only serve as a fluid barrier for the metal layers but also provide crucial design protection against corrosion and abrasion of the subsequent metal components in the structure. Consequently, the premature failure of the polymeric barrier layer during pipeline service can result in substantial costs and pose potential environmental hazards, emphasizing the critical need for its integrity in the flexible pipeline design process [3,4,5,6].

Apart from mechanical integrity, maintaining the characteristics of the polymer layers, particularly their performance related to gas and liquid permeation, is equally important. This is significant due to the presence of typical fluids such as oil, water, H_2_S, CH_4_, or CO_2_, which may commonly be encountered in offshore production [6].

Among the construction polymers utilized in the oil and gas (O&G) industries, polyamide (PA, [(CH_2_)_x_CONH]_n_) stand out as a semi-crystalline thermoplastic extensively employed in various applications. It is commonly used in external anticorrosion coatings for flexible pipes, umbilicals and their accessories, valves, and even as a coating for parts with complex geometries designed for different operating conditions [6]. PA exhibits unique physical properties and good chemical resistance, serving as an effective barrier material during the transportation of gas, water, and oil mixtures for specific scenarios, given that oxidation and/or hydrolysis degradation processes are duly controlled [6].

Hydrolysis is recognized as the primary degradation pathway in humid and acidic environments, where water molecules catalyze the cleavage of amide (C–N) bonds along the polymer backbone. This process results in a reduction of the polymer’s molecular weight and, consequently, in a deterioration of mechanical properties such as tensile strength and elongation at break. Monitoring molecular weight is thus essential in assessing the service life of PA-based systems. The most common method used for this purpose is the determination of the inherent viscosity (IV) in sulfuric acid, which correlates with average molecular weight. The industry-standard API 17 TR2 guideline relies on CIV (corrected inherent viscosity) values to classify material suitability and define safe operational envelopes based on long-term laboratory aging and mechanical test results.

Beyond hydrolysis, interactions of PA with chemicals used in offshore production and transportation can also induce the degradation of the polymer. This is particularly relevant in scenarios where subsea injection of chemicals, including H_2_S-SCVs, is crucial. Consequently, when assessing the service life of PA pressure sheaths, global oil producers and flexible pipe manufacturers commonly monitor corrected inherent viscosity (CIV), following the API 17 TR2 methodology.

However, CIV-based assessments focus solely on molecular weight and may overlook other degradation mechanisms that do not involve significant chain scission. For example, chain crosslinking, microstructural rearrangements, or increased dynamic constraints in the amorphous phase can compromise material performance without necessarily reducing molecular weight. These limitations highlight the need for complementary diagnostic methods capable of detecting subtler or alternative degradation effects.

While there is a reasonable number of works employing spectroscopic techniques to elucidate the structure of H_2_S-SCV [7,8,9], only a few delve into changes in the microstructure and chain dynamics of the polymers exposed to these fluids [5,10]. Among them is our previous study, where ^1^H time-domain nuclear magnetic resonance (^1^H TD-NMR) methods were applied to investigate the effects of H_2_S-SCV exposure on the microstructure and dynamics of PA chains [10]. TD-NMR is a non-destructive, sensitive, and relatively low-cost technique that probes molecular mobility and segmental dynamics, thus offering insights into microstructural and morphological changes at the molecular level. These studies indicated that exposure to H_2_S-SCVs introduces mobility constraints on PA chains within the amorphous phase, shifting the onset of molecular motions associated with the glass transition to higher temperatures. Furthermore, ^1^H DQ-TDNMR experiments in the polymer’s melt state revealed an increase in the density of constrained points in the polymer chains and a decrease in the amount of highly mobile segments upon exposure to H_2_S-SCVs. A specific analysis of the data considering a power law correlation function for the segmental dynamics [11] suggests a scenario with the prevalence of fixed constraining points, as is consistent with the crosslinking of PA chains due to interactions with H_2_S-SCVs. Furthermore, these results were not only correlated to more macroscopic changes observed in CIV and differential scanning calorimetry (DSC) measurements but were also capable of detecting microstructural and dynamical alterations not captured by CIV or DSC measurements.

Despite these findings, several crucial aspects remain unclear, including the effect of exposure temperature, the type of H_2_S-SCV, the mixture of H_2_S-SCVs with other production fluids used in O&G production, and, importantly, a more specific molecular characterization and its relationship with the change observed in the microstructure and chain mobility.

In this article, we conducted a comprehensive characterization of the long-term exposure of commercial PA to H_2_S-SCVs (up to 60 days at 40 °C), with a primary focus on the molecular origin of the microstructure changes. To achieve this, infrared and Raman optical spectroscopy, ^13^C solid-state NMR, and ^1^H time-domain nuclear magnetic resonance (^1^H TD-NMR) were employed and jointly analyzed. In addition, the samples were prepared with a batch of commercial PA and an ethoxylated H_2_S-SCV different from that used in Reference [10].

The ^13^C solid-state NMR, Raman and FTIR spectroscopic results demonstrate that long-term contact with these scavengers leads to the exudation of plasticizers from the PA matrix. This phenomenon explains the observed shift to higher temperatures in the onset of molecular motions related to the glass transition in samples exposed to H_2_S scavengers, as revealed by ^1^H TD-NMR. The new analysis of the ^1^H DQ-TDNMR results confirmed the progressive PA increase in the density of dynamically constrained chains (previously suggested as crosslinking points in [10]) with the exposure to the H_2_S-SCV. Furthermore, the ^1^H DQ-TDNMR experiments in the new set of the samples were designed to allow for a distinct analysis, providing information on the heterogeneity of the dynamic constrains along the samples. A clear trend of increasing heterogeneity over time of exposure was observed. Additionally, the presence of chemical groups of the H_2_S scavenger immobilized in the polymer matrix was found by ^13^C solid-state NMR, thus supporting the idea that the fixed dynamic constrains observed by ^1^H DQ-TDNMR are associated with PA chain crosslinks, and it also suggests that the H_2_S-SCVs play a role in the crosslinking process. These findings suggest that both the formation of crosslinks and the loss of plasticizers contribute to the observed rise in glass transition temperature and the reduction in chain mobility in the molten state, consistent with the formation of a new crystalline phase, as observed in the DSC results of [10] and the gelation of the samples in meta-cresol, which impeded the VIC measurement in samples exposed to H_2_S-SCVs. Furthermore, because the TD-NMR results depicted the same behavior as reported in Reference [10], even though a different H_2_S-SCV and new batch of PA samples were used, it is possible to suggests that this behavior can be generalized to other types of ethoxylated scavengers.

## 2. Materials and Methods

**Samples:** Typical flexible pipe-grade PA was used. The PA samples were cut into cylindrical pieces, with a 4 mm diameter, for ^13^C solid-state NMR and 1 H TD-NMR measurements. Samples of non-exposed PA and PA exposed to pure H_2_S-SCV for 5, 15, 45, and 60 days at 40 °C were studied. The identification of samples was presented as PA-# days, where **#** is the number of exposure days. The maximum exposure time of 60 days was chosen based on preliminary observations indicating that very little additional change occurred beyond 60 days. The exposure temperature of 40 °C was chosen to minimize the risk of hydrolysis, which is known to occur in polyamides at temperatures above 60 °C, even in the presence of trace amounts of water [12,13,14]. To avoid the overlapping of these distinct degradation processes, we chose to perform the exposure at 40 °C, where hydrolysis is significantly less likely to occur. This allowed us to isolate the effects of the H_2_S scavenger on the microstructure of PA11. Attempts to determine the corrected inherent viscosity (CIV) were made for all samples, following the API 17 TR2 methodology, using the same procedure as described in Reference [10]. In this procedure, before the inherent viscosity determination, samples must be solubilized in meta-cresol at 100 °C for 1 h. However, samples that were in contact with pure H_2_S-SCV from this study did not solubilize in this solvent under standard conditions, and thus their CIV could not be measured. Thus, it was only possible to measure the CIV for the PA sample not exposed to the H_2_S; its CIV was found to be 1.6 ± 0.1 dL/g.

**Figure 1:**
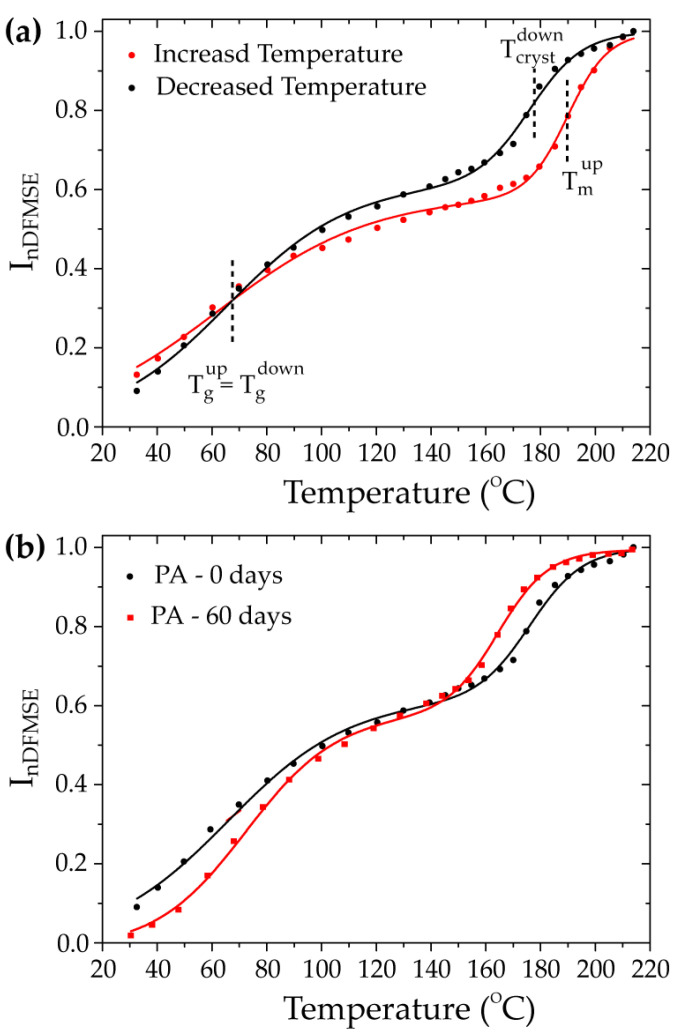
$I_{nDFMSE}\left(T\right)$ vs.  T curves measured for several PA samples. (**a**) Comparison between $I_{nDFMSE}\left(T\right)$ vs.  T curves of non-exposed PA samples acquired at increasing and decreasing temperatures. (**b**) Comparison between $I_{nDFMSE}\left(T\right)$ vs.  T curves of PA samples not exposed to the H_2_S-SCV for 60 days at 40 °C, acquired at decreasing temperatures.

**NMR experiments:** Low-field ^1^H TD-NMR experiments were performed in a 0.5 T Bruker Minispec mq20 NMR analyzer (^1^H frequency of 20 MHz), using a VT probe head with dead time of 12.5 μs. π/2 and π pulse lengths of 2.5 and 4.8 µs, respectively, and recycle delays of 2 s were used. Dipolar-filtered MSE (DF-MSE) pulse sequence (see Figure 1 and References [10,15,16]) were performed with MSE echo times of 100 μs and a dipolar filter time of 40 μs. The variable temperature DF-MSE measurements were performed in a multi-step form, using 10 °C temperature steps and a stabilization period of 10 min. Temperature variation comprised an ascendant range from 20 °C to 200 °C, followed by a descendant range from 200 °C to 20 °C. ^1^H DQ-TDNMR experiments were carried out using double-quantum evolution times, ranging from 0.2 to 10 ms. The data was acquired using the pulse sequence and phase cycling described in Reference [17]. A total of 128 scans were acquired in all experiments. ^1^H DQ-TDNMR measurements were performed at 200 °C, which is above the melting temperature of PA. To guarantee that the samples were in the melt state during the ^1^H DQ-TDNMR measurements, mixed-MSE was performed at 200 °C, showing no sign of rigid components in the samples. ^1^H DQ-TDNMR is being extensively used for probing the presence of crosslinks and entanglements in polymer networks, being an alternative to solvent-base swelling techniques, with many comparisons between crosslink density measure by swelling and ^1^H DQ-TDNMR available in the literature [17,18,19].

**Fourier-Transform Infrared Spectroscopy (FTIR):** FTIR absorbance experiments were conducted using a Magna-IR 850 Series II-Nicolet Spectrometer in the wavelength range of 4000 cm^−1^ to 15,000 cm^−1^, with a resolution of 8 cm^−1^, on samples with a standardized thickness of approximately 1 mm. The surfaces of the samples were prepared and uniformed using successive abrasions with 400–1200-grit sandpaper. A q uartz beam splitter and a tungsten light bulb were used, along with an InGaAs detector.

**Raman spectroscopy:** Raman spectroscopy experiments were conducted using Ocean Optics QE66 Pro equipment within a wavelength ranging from 200 cm^−1^ to 1800 cm^−1^. Ten scans per experiment were performed using a 780 nm laser with a power output of 1000 mW.

## 3. Results

### 3.1. Effects of the H_2_S-SCVs on the Microstructure and Dynamics of PA

To obtain information about overall changes in the PA chain mobility induced by the exposure to the H_2_S-SCVs, we employed a ^1^H TD-NMR experiment known as DF-MSE, which combines the Goldman–Shen dipolar filter (DF) and mixed-MSE. Details of this experiment are described in References [10,16], and its application to probe mobility transitions in PA is explained in Reference [10].

In general terms, this experiment yields a normalized signal intensity, $I_{nDFMSE}$, which accounts exclusively for mobile molecular segments—segments that undergo motion with rates higher than that of the 1H-1H magnetic dipolar coupling, typically in the order of 50 kHz. Therefore, by monitoring the $I_{nDFMSE}$ as a function of temperature, it becomes possible to directly detect the onset temperature of molecular mobility processes through the increase in $I_{nDFMSE}$. This is illustrated in Figure 1a, which shows $I_{nDFMSE}\;vs.\;\;T\;$for PA not exposed to the H_2_S-SCV (PA—0 days).

As observed, there are two main intensity upturns on the $I_{nDFMSE}$ intensity as a function of temperature, associated with the glass transition and the melting of the PA chains. Note, however, that the time scale of the motions detected as mobility is about three orders of magnitude faster than those associated with typical DSC transitions. Consequently, the DF-MSE transition temperature is typically 40 °C higher than the typical temperature expected by DSC for the same process [20].

Typically, each intensity upturn, *i*, in the $I_{nDFMSE}$ vs. T can be well represented by a sigmodal curve, with the temperature at the inflection point denoted by $T_{i}^{1/2}$, and the half-width of the transition region given by $\sigma_{i}$. Thus, $T_{i}^{1/2}$ can be considered the average transition temperature, and $\sigma_{i}$ is somewhat related to the heterogeneity of the transition. Under these assumptions, one can fit the $I_{nDFMSE}\;vs.\;\;T\;$curve for PA, shown in Figure 1, assuming a sum of two sigmodal curves weighted by a fraction, $f_{i}$, to obtain the glass transition and melting temperatures, as well as the corresponding widths and the weights, as seen in the DFMSE experiment. The values of the parameters $T_{i}^{1/2}$, $\sigma_{i}$, and $f_{i}\;$obtained from this fitting are shown in Table 1, where i=1 refers to the mobility transition associated with the glass transition, and i=2 refers to the transition associated with the melting of the sample.

As depicted in Figure 1a and summarized in Table 1, the $I_{nDFMSE}\;vs.\;T\;$curves exhibit differences when acquired with increasing or decreasing temperatures. Notably, the primary distinction lies in the temperature of the high-temperature transition, which is associated with the melting (increasing temperature) and crystallization (decreasing temperature) of PA. This is an expected behavior which happens due to the erasure of the thermal history of the polymer and because the crystallization temperature and fraction (degree of crystallinity) are dependent on the temperature-decrease rate. In addition, as anticipated, the transition temperature associated with the glass transition is about the same for the curves obtained at increasing and decreasing temperatures. The only observed difference is a less spread transition temperature, indicative of more homogeneous dynamics across the sample.

Taking into account the preceding discussion, to detect the effect of the exposure to H_2_S-SCV, we conducted DF-MSE measurements at descending temperatures. Specifically, we initiated the measurements from the molten state of PA, progressively decreasing the temperature at 10 °C/min, with stops of 10 min for temperature equilibration and 10 min for measurement at constant temperature. By starting from the melt state and performing the same temperature-decreasing rate and measurement routine, the $I_{nDFMSE}\;vs.\;\;T\;$curves should only reflect permanent changes in the mobility transitions induced by the H_2_S-SCV.

**Figure 2:**
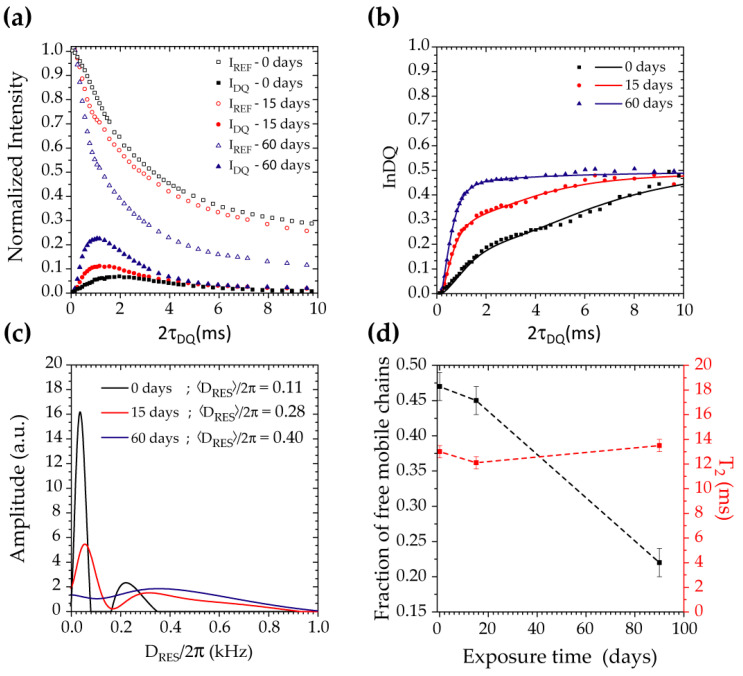
(**a**) $I_{REF}\;\left(\tau_{DQ}\right)$ $vs.{\;\tau }_{DQ}$ and $I_{DQ}\;\left(\tau_{DQ}\right)$ $vs.\;\;\tau_{DQ}$ curve measured with Baum and Pines pulse sequence [17,21] for PA exposed to H_2_S-SCV for several days. (**b**) $I_{nDQ}\;\left(\tau_{DQ}\right)$ $vs.\;\;\tau_{DQ}$ curve obtained from the data shown in (**a**), using the procedure described in the text and Equation (1). (**c**) Distribution of residual dipolar coupling, $D_{RES}$, obtained from the FITREG program described in References [18,19]. (**d**) Fraction of free mobile chains and corresponding T_2_ values obtained from the data shown in (**a**), using the procedure described in the text.

Figure 2b shows a comparison between the $I_{nDFMSE}\;vs.\;\;T\;$curves of the PA not exposed and exposed to the H_2_S-SCV at 40 °C for 60 days (PA—60 days). As can be observed in Figure 1 and Table 1, the sample exposed to the H_2_S-SCV clearly shows an increase in the transition temperature associated with the glass transition, indicating a higher mobility restriction during this process. This is the same behavior observed in Reference [10], but here we used a new batch of PA, a different ethoxylated H_2_S-SCV, and the curves here acquired for pre-molten samples. Thus, the increase in the lower temperature transition on the $I_{nDFMSE}\;vs.\;\;T\;$curves confirms that the exposure to ethoxylated H_2_S-SCV induces a permanent modification in the mobility behavior during the glass transition of the PA chains.

^1^H DQ-TDNMR experiments were performed using the Baum and Pines pulse sequence described in Reference [21]. The first aim of these experiments was to confirm in this set of samples our previous finding that exposure to the H_2_S-SCV induces permanent mobility restrictions that could be detected in the melt state of the samples [10]. Thus, to guarantee that all samples would be melted and have similar overall mobilities, the DF-MSE curves were used to determine the temperature for the 1H DQ-TDNMR experiments, and the temperature was found to be 210 °C. This is clearly observed in Figure 2a, which depicts the double-quantum intensities, $I_{DQ}$, and the reference intensities, $I_{REF}$, as a function of the experimentally adjusted periods, $\tau_{DQ}$. Details on the 1H DQ-TDNMR signal acquisition and the meaning of the $I_{DQ}\;vs.{\;\tau }_{DQ}\;$and $I_{REF}\;vs.\;\tau_{DQ}\;$can be found in Reference [10]. Here, we just state the main features. Both intensities are determined from time-domain NMR signals by applying the pulse sequence described in Reference [17]. This pulse sequence encodes the double-quantum intensities, $I_{DQ}$, upon the evolution of ^1^H nuclear spins in the presence of the so-called ^1^H-^1^H magnetic dipolar coupling. On the other hand, ^1^H-^1^H dipolar coupling depends on the orientation of the ^1^H-^1^H spin pairs in respect to the external magnetic field, and the average of this interaction become zero in the presence of fast isotropic mobile segments. This means that isotropic mobile segments do not generate double quantum coherences, so they do not contribute to $I_{DQ}$, which can only build up from segments that present any kind of mobility restriction. Thus, in a polymer melt, the $I_{DQ}$ would be directly related to the presence of mobility restrictions associated either with chain entanglements or crosslinks. Moreover, the build-up rate of $I_{DQ}$ with $\tau_{DQ}$ is directly related to the residual dipolar coupling $\left(D_{RES}\right)$ between ^1^H nuclear spins in segments with mobility restriction. Because $D_{RES}$ is inversely proportional to the cube of distance between the nuclear spins, it becomes proportional to the density of points experiencing mobility restrictions, directly translating to the density of entanglements or crosslinks in the polymer. Thus, the $I_{DQ}$ build-up curve ($I_{DQ}\;vs.\;\tau_{DQ}$) comprises an initial slope proportional to the density of entanglements/crosslinks. At a longer $\tau_{DQ}$, the $I_{DQ}\;vs.\;\;\tau_{DQ}$ curve decays due to transverse relaxation (T_2_), so the initial build-up is followed by an exponential-like decay, as observed in the curves shown in Figure 2a. In addition to that, the $I_{REF}$ intensity arises from all molecular segments and only decay due to the T_2_ relaxation. An interesting point is that the remaining intensities, $I_{REF}$, for $\tau_{DQ}$ values where $I_{DQ}$ has already decayed will be proportional to segments that do not experience mobility constraints, i.e., free mobile segments. Thus, the long $\tau_{DQ}$ tail of the $I_{REF}\;vs.\;\;\tau_{DQ}$ curve is only dependent on the free mobile chains.

The $I_{DQ}\;vs.\;\tau_{DQ}$ and $I_{REF}\;vs.\;\tau_{DQ}$ curves for PA not exposed and exposed to the H2S-SCV at 40 °C for 15 and 60 days are shown in Figure 2a. They depict the same behavior as reported previously in [10], showing an increasing slope of $I_{DQ}\;vs.\;{\;\tau }_{DQ}$ and a decreasing intensity of the long $\tau_{DQ}$ tail $I_{REF}\;vs.\;\;\tau_{DQ}$. This was interpreted as evidence that the H_2_S-SCV induced permanent mobility restrictions to the PA chains and reduced the number of free mobile ones. Indeed, an analysis of these functions using prober fitting function based on the motion correlation function lead to the conclusion that these mobility restrictions are likely associated with crosslinks. Therefore, our new set of measurements, for a new batch of PA and other types of ethoxylated H_2_S-SCV, reproduce the previously observed behavior very well.

As mentioned, from the analysis based on the motion correlation function, presented in References [10,11], we already know that the H_2_S-SCV-induced mobility restrictions are likely to be due to chain crosslinks. Thus, we perform here a different analysis of the $I_{DQ}\;vs.\;\tau_{DQ}$ and $I_{REF}\;vs.\;\tau_{DQ}$ curves. This analysis in quite usual in the context of elastomeric networks, were $I_{DQ}$ is intricately linked to crosslinked and/or entangled chains. The first part of the procedure consists on subtracting$\;I_{REF}\;vs.\;\tau_{DQ}$ and $I_{DQ}\;vs.\;\tau_{DQ}$ curves to eliminate the contribution of crosslinked/entangled chains and fitting the extended $\tau_{DQ}$ tail of the resulting curve with an exponential function, such as $f_{m}e^{-\frac{{2\tau }_{DQ}}{T_{2m}}}$. As mentioned, at a long $\tau_{DQ}$ the behavior of $I_{REF}\;vs.\;\;\tau_{DQ}$ and, consequently, $\left(I_{REF}-I_{DQ}\right)\;vs.\tau_{DQ}\;$is only associated with free mobile segments. Thus, the amplitude, $f_{m}$, represents the fraction of these segments in the sample, and $T_{2m}$ is the corresponding transverse relaxation time, which is associated with the degree of molecular mobility of these segments. The next step is to use $f_{m}$ and $T_{2m}$ to obtain the so-called normalized double-quantum intensity, $I_{nDQ}$, which only depends on the build-up of the double-quantum coherences, i.e., without contribution from the free mobile segments and relaxation [17,18].

(1) $$I_{nDQ}\left(\tau_{DQ}\right)=\frac{I_{nDQ}\left(\tau_{DQ}\right)}{{I_{REF}\left(\tau_{DQ}\right)+I}_{nDQ}\left(\tau_{DQ}\right){-f}_{m}e^{-\frac{{2\tau }_{DQ}}{T_{2m}}}}$$

While the initial slope of the $I_{nDQ}\left(\tau_{DQ}\right)$ $vs.\;\;\tau_{DQ}\;$curve highlights the information about the residual dipolar coupling proportional to the density of crosslinked/entangled chains, its shape depend on the distribution of $D_{RES}$, bringing also information about the distribution of the average crosslink density. A proper mathematical procedure based on the Tikhonov regularization, named FITREG and described in References [18,19], is then used to provide the $D_{RES}$ distribution curve.

Figure 2b shows the $I_{nDQ}\left(\tau_{DQ}\right)$ $vs.\;\;\tau_{DQ}\;$curves obtained for the PA not exposed and exposed to the H_2_S-SCV at 40 °C for 15 and 60 days. The increase in the initial slope upon the exposure time becomes rather evident. Moreover, the shape of the curves is also changed for samples more exposed to the H_2_S-SCV, suggesting a significant change in the distribution of crosslinks in the sample. The corresponding $D_{RES}$ distribution curves are shown on Figure 2c. The average $\langle D_{RES}\rangle$ values obtained for these curves are present in the legend, showing a rather significant increase, as expected from the slope change.

The changes in the $D_{RES}$ distributions associated with the exposure of the samples to the H_2_S-SCV follow a clear pattern. For the non-exposed sample, the distribution comprises an intense and sharp component that peaks at ~50 Hz and a much less intense component peaking at ~250 Hz. These components are likely to be attributed to entanglement chains in the melt state of even a small portion of the crosslinking present on PA. While this bimodal shape seems to be preserved in the samples exposed to the H_2_S-SCV, there is a clear and progressive increase in the contribution of components with high $D_{RES}$ values to the distribution upon the exposure time. This is again in line with some H_2_S-SCV families promoting the crosslinking of the chains but adds up information in the sense that it shows that it produces a rather inhomogeneous distribution of such crosslinks. This is consistent with the lack of solubility of the exposed H_2_S-SCV samples in meta-cresol, which prevented the measure of the CIV in the samples.

Last, we show the evolution of the fraction of free mobile chains, $f_{m}$, and the corresponding $T_{2m}$ upon the exposure time in Figure 2d. An evident decrease in $f_{m}$ and almost constancy of $T_{2m}$ is observed for increasing exposure time. Indeed, the constancy of $T_{2m}$ is anindicative that, at 205 °C, all samples are in similar mobility regimes.

### 3.2. Changes in the PA Chemical Composition Due to Exposure to the H_2_S-SCVs

In the former section, we confirmed and further characterized the microstructure and mobility changes induced by the H_2_S-SCV in the investigated set of PA samples. We now focus on the possible changes in their chemical composition. To do so, we use two optical techniques, Fourier-Transform Infrared Spectroscopy (FTIR) and Raman spectroscopy, and ^13^C CPMAS high-resolution NMR. All of them offer a detailed analysis of specific molecular groups, allowing them to draw conclusions that are compatible with and complementary to the ^1^H TDNMR results previously described.

**Figure 3:**
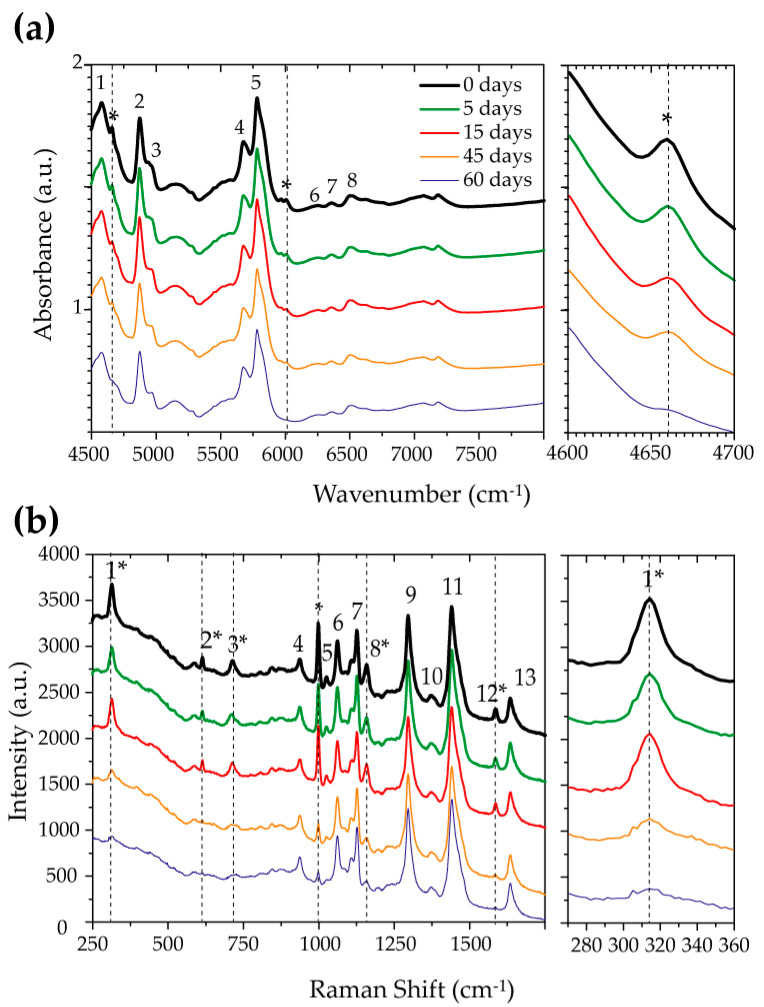
(**a**) Fourier-Transform Infrared Spectroscopy spectra of PA samples not exposed and exposed for 5, 15, 45, and 60 days. The up numbers correspond to the bands assigned in the text. The bands not attributed to PA are marked with an asterisk. The right panel shows a zoom-in of the 4600 to 4700 cm^−1^, corresponding to a band not attributed to PA. (**b**) Raman spectra of PA samples not exposed and exposed for 5, 15, 45, and 60 days. The up-numbers correspond to the bands assigned in the text. The bands not attributed to PA are marked with an asterisk. The right panel shows a zoom-in of 270 to 360 cm^−1^, corresponding to a band of the BBSA plasticizer.

Figure 3a shows the FTIR spectrum of the samples not exposed and exposed to the H_2_S-SCV for 5, 15, 45, and 60 days. The main bands associated with the samples were assigned in Reference [22]. The attribution to the bands numbered in Figure 3a is 1-4583 cm^−1^-ν(NH)_b_ + Amide III; 2-4871 cm^−1^-ν(NH)_b_ + Amide II; 3-4969 cm^−1^-ν(NH)b + Amide I; 4-5677 cm^−1^-2⋅νs(CH2); 5-5784 cm^−1^-2⋅νaδ(CH2); 6-6256 cm^−1^-Amide I + II) + Amide B; 7-6368 cm^−1^-ν(NH)b + Amide B; 8-6502 cm^−1^-2⋅ν(CH2). One should acknowledge that some bands could not be conclusively assigned based solely on the expected results for the polyamide structure. This suggests that these unassigned peaks are likely attributable not to PA chains, but to another chemical compound present in its composition. Considering that the samples are commercial formulations, it is somewhat expected that, besides polyamide, they contain a significant percentage of additives, with the most common being a plasticizer. Indeed, for the formulations used for pipeline building, it is expected for about 10% of the sample to be a plasticizer, with N-butyl benzene sulfonamide (BBSA) being the plasticizer most used [23,24].

Figure 3a also shows that the bands associated to the PA structure do not change in the samples exposed to the H_2_S-SCV. However, there is a clear decrease in the bands at 4659 cm^−1^ and 5966 cm^−1^, as indicated in the figure, with the exposure time. This behavior is highlighted in the lateral panel, which shows a zoom-in of the 4600 cm^−1^ to 4700 cm^−1^ spectral regions. This suggests that the chemical structure of the PA is not affected by the exposure to the H_2_S-SCV, but there is a progressive removal of additives with the exposure time.

To investigate the specific additive removed from the samples due to the exposure to the H_2_S-SCV, we performed Raman spectroscopy measurements, for which the results are shown in Figure 3b. Raman spectra of the PA and the plasticizer BBSA have been already reported in the literature [25]. The main Raman bands shown in Figure 3b are assigned as 1-313 cm^−1^-C-S stretching-BBSA; 2-612-NH bending-BBSA; 3-715-CH bending-BBSA; 4-936-C=O stretching-polyamide 11; 5-1024-CH bending-BBSA; 6-1064-C-C stretching-polyamide 11; 7-1127-C-C stretching-polyamide 11; 8-1158-CH bending-BBSA; 9-1296-C=N stretching-polyamide 11; 10-1376-CH_2_ vibration-polyamide 11; 11-1439-CH2 vibration-polyamide 11; 12-1585-benzene ring stretching-BBSA; and 13-1635-C=O vibration-polyamide 11.

It can be clearly observed in Figure 3b that six of the identified bands underwent changes with increasing exposure time to the H_2_S-SCV. All of these bands, located at wavelengths 313 cm^−1^, 612 cm^−1^, 715 cm^−1^, 1024 cm^−1^, 1158 cm^−1^, and 1585 cm^−1^, are assigned to BBSA. A gradual intensity reduction was observed with prolonged exposure time until a full disappearance in the spectrum of the sample with 60 days of exposure. This is highlighted in the zoomed-in image of the 280 cm^−1^ to 360 cm^−1^ regions in Figure 3b. Therefore, the Raman measurements clearly indicate that prolonged exposure to the H_2_S-SCV promotes the exudation of the BBSA plasticizer.

**Figure 4:**
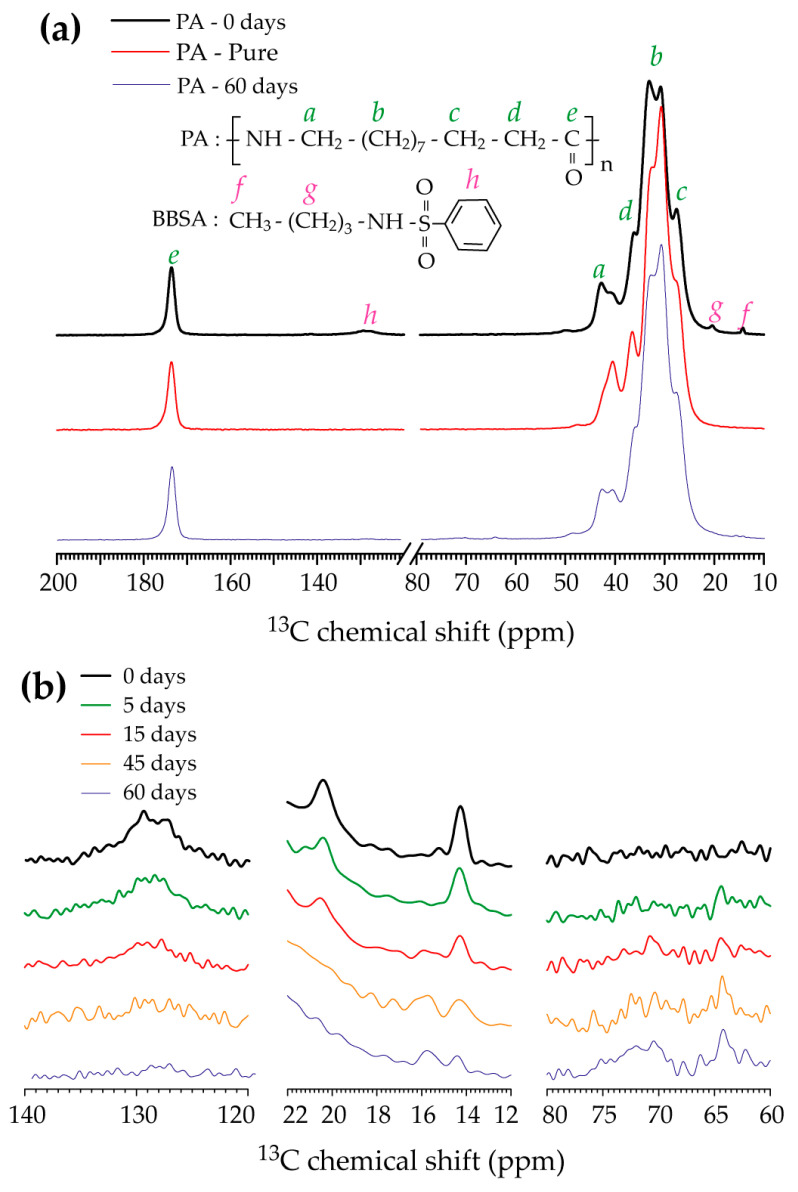
(**a**) ^13^C CPMAS solid-state NMR spectra of polyamides 11 with the mains signals assigned according to the chemical structure of PA and BBSA plasticizer shown as inset. Top, PA—0 days (commercial PA); middle, PA—pure PA, without additives; bottom, PA—60 days (commercial PA exposed to H_2_S-SCV for 60 days). (**b**) Selected spectral regions of the ^13^C CPMAS spectra of PA exposed to H_2_S-SCV for the time periods indicated.

Further evidence of the BBSA plasticizer’s exudation due to the exposure to the H_2_S-SCV was obtained from the ^13^C CPMAS s olid-s tate NMR spectra shown in Figure 4. The spectra of the PA—0 days exhibited in Figure 4a—show all main signals expected for PA (indicated by the green letters), along with some minor signals that can be attributed to the carbons of the BBSA plasticizer (indicated as magenta letters). This was confirmed by comparing the spectra with that of an analytical-grade PA sample (pure PA, not plasticized), also shown in Figure 4a. Those differences in the intensity pattern of the main PA signals can be attributed to differences in the structure of the crystalline phases in each sample [26,27]. However, it is also clear that the signals attributed to the BBSA plasticizer are not present in the spectra of the pure PA sample.

The signals attributed to the BBSA plasticizer are clearly reduced in the ^13^C CPMAS solid-state NMR spectra of the PA—60 days. Besides that, some extra signals also show up in the 60–80 ppm region. This is better observed in Figure 4b, which shows a zoomed-in image of selected spectral regions of the ^13^C CPMAS spectra of PA samples exposed to H_2_S-SCV for different time periods. The diminishing of the signals due to the BBSA plasticizer at increasing exposure time is evident from the left and middle panels in Figure 4b, in complete agreement with the results obtained from FTIR and Raman spectroscopies. Moreover, the ^13^C CPMAS spectra of the samples also show the progressive appearance of some signals in the 60–80 ppm region shown in the right panel of Figure 4b. A common characteristic of all H_2_S-SCVs used in the O&G industry is that they may be ethoxylated, which means that there would be signals in the 60–80 ppm region. Thus, the ^13^C CPMAS spectra suggest that some chemical groups associated with the H_2_S-SCV are incorporated in the samples. Moreover, ^1^H-^13^C cross-polarization excitation provides a type of slow mobility filter in the sense that signals from free isotropic mobile molecules do not show up. Thus, the appearance of signals from typical H_2_S-SCV groups in the ^13^C CPMAS spectra suggests that these groups are somewhat immobilized.

## 4. Discussions

After establishing the primary changes in chemical composition, microstructure, and chain mobility induced by the exposure of PA to H_2_S-SCVs, it is crucial to explore the interrelationships among these features.

The removal of plasticizers from polyamide due to exposure to fluids, especially in offshore oil and gas environments, has been shown to significantly impact the polymer’s properties [28,29,30,31]. Studies indicate that fluid contact, particularly at elevated temperatures, accelerates plasticizer exudation from the PA matrix, leading to increased stiffness, and embrittlement [32]. For example, exposure to oilfield water and chemical additives can promote plasticizer loss, which in turn raises the glass transition temperature (Tg) and reduces chain mobility, adversely affecting mechanical performance [32].

The removal of the BBSA plasticizer in the studied samples was confirmed through FTIR, Raman, and ^13^C solid-state NMR spectroscopy. Plasticizer additives typically serve to facilitate processing by filling spaces between polymer chains, reducing their interaction, and consequently increasing their mobility [23]. Thus, the elimination of the BBSA plasticizer due to the exposure of PA to the H_2_S-SCV appears to be directly associated with the observed increase in the glass transition temperature in the DF-MSE experiments, as shown in Figure 1b and Reference [10]. Additionally, since the plasticizer is a small molecule not chemically bound to PA, it should behave as free mobile segments at temperatures above the melting temperature of PA. Thus, the decrease in the fraction of free mobile segments observed in the ^1^H DQ-TDNMR results shown in Figure 2 points to the same direction. It is worth mentioning that thermal and viscometric analysis probes bulk phenomena with no molecular resolution, while optical spectroscopy gives atomic-level information, but mostly associated with the sample’s surface due to the reduced penetration depth of the light (for transmission/absorption measurements the thickness of the samples must be reduced). On the other hand, ^13^C ssNMR spectroscopy probes the sample bulk with atomic resolution, which can be complementary to optical spectroscopy, and time-domain NMR provides specific dynamics information that can be correlated with the thermal measurements. Thus, the NMR measurements suggest that the plasticizer exudation is not restricted to the sample surface and imply motional hindrance of the PA chain motions that may lead to changes in the thermomechanical properties. A more specific analysis on the influence of microstructural change on the mechanical properties is needed.

Thus, our results indicate that the exposure to ethoxylated H_2_S-SCVs can promote the exudation of the PA plasticizer even at a low temperature (40 °C), which is in line with Reference [32], indicating that it can lead to the same kind of changes in the mechanical properties of the PA samples. Because in offshore oil and gas environments, multiple types of fluids are used, the uses of H_2_S-SCVs’ cocktails will add up a source that promotes the exudation of the PA plasticizer.

The ^1^H DQ-TDNMR experiments demonstrated the increase in the density of dynamically hindered segments in the melt state of PA exposed to the H_2_S-SCVs. Thus, the presence of rigidified segments of the H_2_S-SCVs, observed by ^13^C CPMAS, adds another piece of evidence that this dynamic restriction is related to chemical crosslinks induced by the H_2_S-SCVs, suggesting that the H_2_S-SCV itself may act as a crosslinking agent.

It is known that long-term exposure of polyamide 11 (PA11) to different fluids in extreme environmental conditions can induce significant chemical degradation, primarily through chain scission or crosslinking. Chain scission results in a decrease in molecular weight and intrinsic viscosity, directly compromising mechanical strength and ductility. Mazan and co-workers demonstrated that a reduction in intrinsic viscosity correlates with diminished mechanical properties in degraded PA11 samples [29]. Other studies explored the embrittlement processes in polyamide 11 (PA11) during thermal oxidation. It was suggested that while chain scission is a primary degradation mechanism, crosslinking can also occur, leading to increased brittleness. This study emphasizes that embrittlement is influenced by both molecular weight reduction and changes in crystalline morphology, particularly the interlamellar distance [33,34]. This suggests that inhomogeneous crosslinking contributes to the material’s embrittlement over time.

In the ^1^H DQ-TDNMR results presented here, there is also evidence of an increase in the crosslink inhomogeneity with the exposure time of the PA to the H_2_S-SCV, which can impact the mechanical properties of PA. This is evidence of the lack of solubility of the PA exposed to the H_2_S-SCV in meta-cresol.

Finally, the removal of the plasticizer allows PA chains to come into closer proximity, favoring crosslinking reactions involving the H_2_S-SCV. This provides an explanation that links the removal of the plasticizer and the increase in crosslinks suggested by our experimental results.

## 5. Conclusions

This study investigates the molecular and microstructural transformations of commercial polyamide (PA), used as a barrier layer in flexible pipelines for offshore oil and gas production, when exposed to an ethoxylated H_2_S scavenger. A multi-technique analytical approach—comprising FTIR, Raman, ^13^C solid-state NMR, and ^1^H time-domain NMR—was employed.

A key finding was the exudation of the BBSA plasticizer, confirmed by FTIR, Raman, and ^13^C solid-state NMR. This plasticizer loss led to a shift in the glass transition temperature due to reduced molecular mobility, as evidenced by Dipolar-Filtered Magic Sandwich Echo (DF-MSE) time-domain NMR, and a decrease in the fraction of highly mobile segments in the melt state, as observed by ^1^H Double-Quantum (DQ) time-domain NMR—both performed at low magnetic field. These findings are consistent with previous thermal and viscometric measurements and indicate that plasticizer loss is not restricted to the surface but extends throughout the bulk of the material.

The presence of immobilized segments and reduced solubility in meta-cresol further supports the formation of rigid, crosslinked domains, suggesting that the H_2_S-SCV not only promotes plasticizer exudation but may also act as a crosslinking agent. Moreover, ^1^H DQ TDNMR revealed increasing inhomogeneity in the crosslinked network with prolonged exposure. The combined effects of plasticizer removal and crosslink formation likely contribute to the degradation of PA’s mechanical performance, including embrittlement and reduced ductility.

Overall, the results highlight the need to consider the complex interplay between chemical composition, microstructure, and chain dynamics when evaluating the long-term performance of PA-based barriers in environments containing H_2_S-SCVs.

## Figures and Tables

**Table 1:** Parameters extracted from DF-MSE vs. temperature.

Sample	$T_{1}^{\frac{1}{2}}({}^{\circ}C)$	$T_{2}^{\frac{1}{2}}({}^{\circ}C)$	$\sigma_{1}({}^{\circ}C)$	$\sigma_{2}({}^{\circ}C)$	$f_{1}$	$f_{2}$
PA—0 days	66 ± 2	176 ± 1	22 ± 1	10 ± 1	0.62 ± 0.01	0.38 ± 0.01
PA—60 days	74 ± 1	165 ± 1	17 ± 1	9 ± 1	0.59 ± 0.01	0.41 ± 0.01

## Data Availability

Data is available by direct contact with the corresponding author.
